# Insights on the Influence of the Drying Method and Surface Wettability on the Final Properties of Silica Aerogels

**DOI:** 10.3390/gels11070511

**Published:** 2025-07-01

**Authors:** Beatriz Merillas, Maria Inês Roque, Cláudio M. R. Almeida, Miguel Ángel Rodríguez-Pérez, Luisa Durães

**Affiliations:** 1 Cellular Materials Laboratory (CellMat), Condensed Matter Physics Department, Faculty of Science, University of Valladolid, Campus Miguel Delibes, Paseo de Belén 7, 47011 Valladolid, Spain; marrod@uva.es; 2 University of Coimbra, CERES, Department of Chemical Engineering, Rua Sílvio Lima, 3030-790 Coimbra, Portugal; inesroque@eq.uc.pt (M.I.R.); claudio@eq.uc.pt (C.M.R.A.); 3 BioEcoUVA Research Institute on Bioeconomy, University of Valladolid, 47011 Valladolid, Spain; 4 Department of Chemistry, Faculty of Sciences, University of Burgos, 09001 Burgos, Spain

**Keywords:** silica aerogels, freeze drying, supercritical drying, textural properties

## Abstract

In the synthesis of aerogels, the influence of the drying process on the nanostructure is an issue of utmost relevance for tailoring the final properties of these materials. Among the complex parameters affecting this process, the hydrophobicity of the aerogel structure plays a key role. Thus, herein, four different silica aerogel formulations based on tetraethyl orthosilicate and trimethoxymethylsilane were employed to produce aerogels with different wettability properties (from hydrophilic samples to highly hydrophobic). The synthesized gels were dried by three methods, namely freeze-drying, high-temperature supercritical drying with ethanol, and low-temperature supercritical drying with carbon dioxide, and the influence of each procedure on bulk density, porosity, pore size, and specific surface area of the resulting aerogels was analyzed in detail. The direct correlation between the surface hydrophobicity/hydrophilicity of the silica gels and the effects of each drying technique was analyzed, providing insights into a proper selection of the drying method depending on both the water affinity of the gel and the desired textural properties and structures.

## 1. Introduction

Silica aerogels are materials that, since their discovery in 1931 [1,2], have reached growing attention, and the forecast is a continuous increase in their production and scientific research [3]. Their outstanding properties are a direct consequence of their structural features, particles and pores, which present a nanometric size. Thus, silica aerogels have ultra-low densities in the range of 0.03 to 0.3 g/cm^3^ [4,5] that, in comparison to the bulk density of solid silica (2.2 g/cm^3^) [6], are understandable by the presence of a huge porosity with values sometimes higher than 97%. Combining high porosities with nanometric particles also gives rise to high specific surface areas that can reach values over 1200 g/m^2^ [7]. Therefore, these exceptional characteristics lead to unparalleled thermal insulation and optical transparency because of their similar refractive index to air and pores in the nanoscale [8], which, combined with their lightweight, make them highly suitable for advanced glazing and energy-efficient windows [9,10]. Furthermore, their low dielectric constants and high specific surface areas open up innovative applications in electronic devices, adsorption, and catalysis [11,12].

Nevertheless, thorough control of the porous structure and solid skeleton is needed to tailor the derived properties of aerogels [13]. For this reason, different approaches have been followed in the literature. For instance, modifying the synthesis parameters such as silica precursors [14,15], concentrations, solvents [16], pH [17], catalysts’ amount or nature [18], or aging time [19] are frequent strategies for modulating the final structures of the silica aerogels [20]. Moreover, other techniques such as mechanical reinforcement with fibers [21], particles [22], scaffolds [23,24,25], or polymer crosslinking [26,27], or physical modulation of the pore size through compression [28,29] are also alternatives to reach the desired properties in aerogels. Despite these techniques facilitating the control of the silica aerogel structures, it has been widely studied that the drying method employed for extracting the solvent used during the synthesis also presents a critical influence on the aerogel structures and, therefore, properties [30]. In general terms, ambient pressure drying leads to aerogels with a higher density and reduced porosity as a result of strong shrinkages [31]. These shrinkages are promoted by the capillary forces acting during evaporation of the solvent and condensation reactions of silanol groups, which result in irreversible shrinkages and pore collapse [32,33]. Nevertheless, the effect of these forces can be reduced by three methods: (i) by exchanging the polar solvent with a low-surface-tension solvent (apolar), as investigated by Maleki et al. [34]; (ii) by enhancing the mechanical stability of the gel through strengthening the solid network for better withstanding forces; or (iii) by decreasing the presence of hydroxyl groups on the gel surface, that is, by increasing hydrophobicity, to reach the so-called springback effect (SBE) in which the gel undergoes a re-expansion after an initial shrinkage [35]. This last effect is usually observed in samples with non-polar endgroups. Thus, it is common to perform a surface treatment with silylating agents such as trimethylchlorosilane (TMCS), as reported in the works by Khedkar et al. [36,37], or hexamethyldisilazane (HMDZ) for promoting hydrophobicity in the aerogel system and the subsequent shrinkage decrease during the drying procedure [38,39].

Furthermore, each drying method presents crucial parameters that also determine the microstructure control. Such parameters include the drying temperature in ambient pressure drying, the working pressure and duration in supercritical drying [40,41], or the freezing rate in freeze-drying [42]. Focusing on supercritical drying, drying media also affect the surface properties of the obtained samples, since supercritical drying with hot alcohols usually leads to hydrophobic aerogels, as their surface becomes covered by the corresponding alkyl groups through re-esterification, whereas supercritical carbon dioxide extraction provides hydrophilic aerogels [43].

Thus, the chemical nature of the gel skeleton, governed by the surface functional groups, plays a significant role in the nanoporous structures that will determine the final properties. Owing to the complexity of the influence of drying procedures in textural properties, there is a need to understand how to select the proper drying method depending on the aerogel’s water affinity to obtain the optimum properties regarding density, specific surface areas, and porosity for the desired applications. Although other works in the literature address possible solutions for reducing shrinkage during drying and suggest a shift to cost-effective drying methods such as ambient pressure drying while keeping acceptable densities and porosities in the final materials, there is a gap in the understanding of the role that the surface characteristics play in material restructuring during different drying protocols.

Therefore, in this work, the authors selected silica aerogel systems covering different levels of hydrophobicity through modifications in the precursor concentrations (TEOS and MTMS) and dried the samples by three different procedures: freeze-drying, high-temperature supercritical drying (with alcohol), and low-temperature supercritical drying (with CO_2_). Then, the effect of each process on their structural/textural properties was studied in detail, providing effective insights into the relationship between the water affinity properties and the influence of each drying method on their porous structures. Thorough control of the structural features is crucial for obtaining materials with enhanced performance for specific applications.

## 2. Results and Discussion

### 2.1. Silica Aerogel Samples and Hydrophobicity

**Figure 1:**
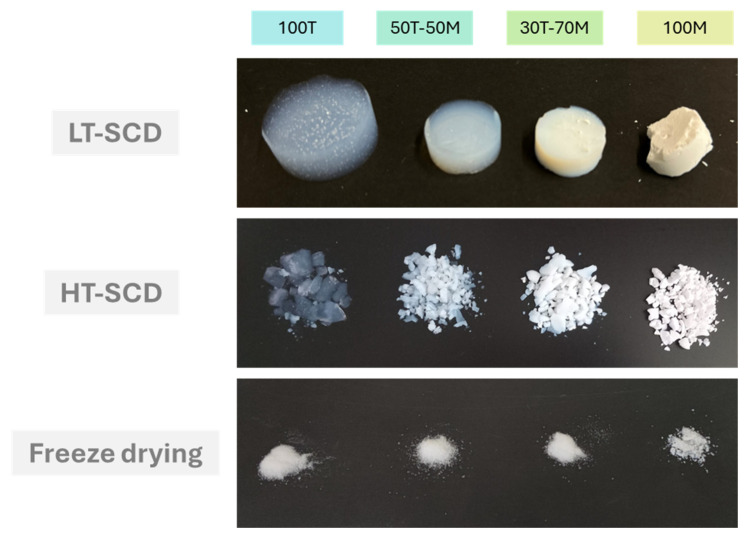
Aerogel samples after the different drying processes.

The four different gel formulations were dried with the three methods described in Section 4.3, resulting in 12 different silica aerogel samples, as can be seen in Figure 1. The choice of TEOS and MTMS as precursors was based on their chemical nature, which provides wettability differences owing to the controlled balance of hydroxyl and methyl moieties. While TEOS presented four hydroxyl groups for condensation, MTMS had a “locked” position by a methyl group, yielding a higher hydrophobic degree and fewer linking points. At first glance, differences in the appearance of all the aerogels were observed. The aerogels dried by LT-SCD preserved their original shape as a result of the appeased capillary forces present during the ethanol extraction by supercritical CO_2_ (scCO_2_). On the other hand, gels dried by HT-SCD were broken into small pieces, and the aerogels that were dried by freeze-drying were collected in powder form as a consequence of the mechanical stress caused by the increase in the specific volume of water in the pores during freezing. Thus, the mechanical integrity of these samples was better maintained when dried by low-temperature supercritical drying, independently of their surface chemical nature.

**Figure 2:**
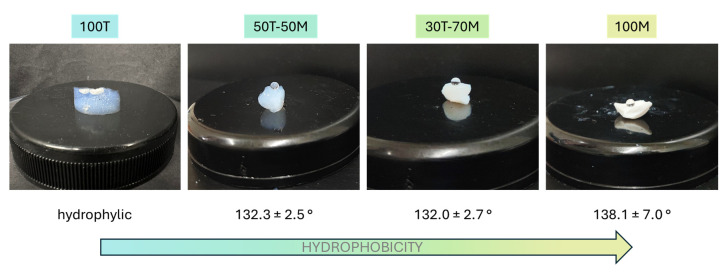
Contact angles for all the aerogel samples under study.

In order to understand the influence of the drying methods on each formulation, the hydrophilic properties of the produced samples (those dried by LT-SCD) had to be measured, since a strong correlation between water affinity and the drying principle was expected (no re-esterification). In Figure 2, it can be seen that the 100T sample presents a hydrophilic nature, whereas the contact angles for the rest of aerogels vary between 132 and 138° (all considered hydrophobic materials according to the IUPAC classification), showing a direct trend with the type of silica precursors; 100T presents the most hydrophilic behavior because of the hydroxyl groups present on its surface, whereas when including a 50% or 70% mol. of Si from MTMS precursor, methyl groups are present in the structure, significantly increasing the hydrophobic behavior, but with no remarkable differences between the latter. Finally, the most hydrophobic sample was produced with 100% of MTMS.

### 2.2. Effect of Drying on Density and Porosity

All the samples were characterized in terms of density, porosity, specific surface area, and mean pore size. The results obtained are gathered in Table 1. The varying proportions of each precursor resulted in different chemical compositions, in which the presence of typical chemical bonds—commonly reported in similar materials by FT-IR—was to be expected [44]. The skeletal densities obtained by helium pycnometry and used for the calculations were 1.645 g/cm^3^ for 100T, 1.353 g/cm^3^ for 50T-50M, 1.409 g/cm^3^ for 30T-30M, and 1.315 g/cm^3^ for 100M.

**Figure 3:**
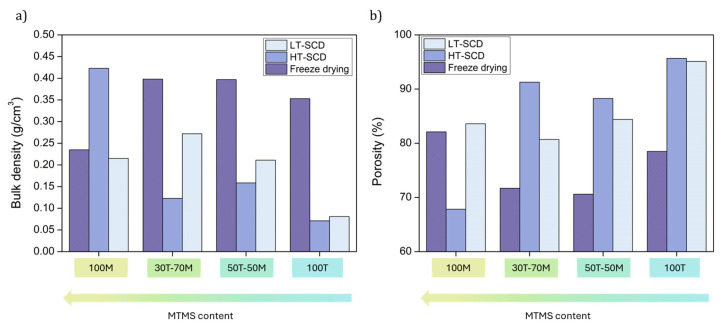
(**a**) Bulk density and (**b**) porosities of all the aerogel samples.

The values obtained for bulk density and porosity are plotted in Figure 3 for an enhanced visualization. There exists a clear influence of the drying method on the sample density and, therefore, porosity. In general, the freeze-drying method provided the highest densities (and lowest porosities) as a result of a strong shrinkage associated with the presence of ice microcrystals [45]. This occurred for all the aerogels except the most hydrophobic ones, the 100M sample, which presented a similar density to that obtained by LT-SCD. This can be explained by the higher hydrophobic surface of that sample, which reduces the interaction with water during the freezing and lyophilization processes and may result in a structure less damaged by the ice crystals. Thus, freeze-drying led to more similar results in density and porosity than those obtained by SCD for samples with a more hydrophobic surface. The porosities reached by freeze-drying for these samples were between 70.6 and 82.1% (see Figure 3b). However, when inducing a higher hydrophilicity in the silica aerogels, the differences between this method and supercritical drying were more remarkable.

On the other hand, when comparing supercritical methods, it can be stated that the HT-SCD method led to lower densities than LT-SCD for higher TEOS contents, probably due to the re-esterification phenomenon that produces hydrophobic pore surfaces [46]. The density of the 100MTMS sample was significantly higher when dried by HT-SCD; that is, for hydrophilic samples, HT-SCD led to nanoporous structures with high porosities, whereas when aerogels were more hydrophobic (100 MTMS), the low-temperature process with sc-CO_2_ was a more effective drying technique in preserving the gel structure.

### 2.3. Effect of Drying on the Textural Properties and Porous Structures

**Figure 4:**
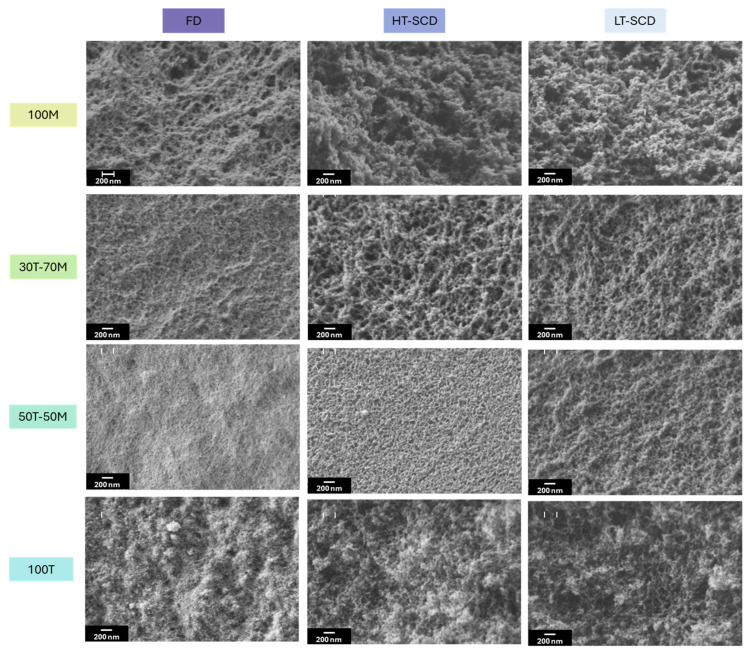
Scanning electron micrographs at the same magnification for all the formulations and drying methods investigated in this work.

**Figure 5:**
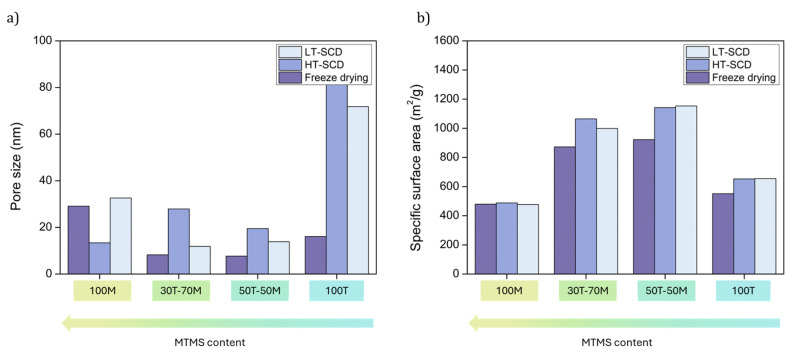
(**a**) Mean pore size and (**b**) specific surface area (*S*_BET_) of all the aerogels obtained with the different drying procedures.

Once the influence of the drying procedure on density and porosity was explored, the effect of the drying methods on the nanoporous structures and textural properties should be studied. The scanning electron micrographs are gathered in Figure 4. As a consequence of the clear densification of the particulate skeleton when drying the aerogels by freeze-drying, especially for 30T-70M, 50T-50M, and 100T, the pore size was reduced for these samples, as observed in Figure 5a. In fact, the percentage reduction in pore size when drying by freeze-drying in comparison to HT and LT-SCD followed a clear trend; less hydrophobic samples presented a higher pore reduction because of the higher surface energy; for instance, for 100T aerogel, these reductions were by ca. 80% with respect to both SCD techniques. Nevertheless, these differences progressively diminished when inducing hydrophobicity until reaching similar pore size values for 100M in comparison to the LT-SCD.

**Figure 6:**
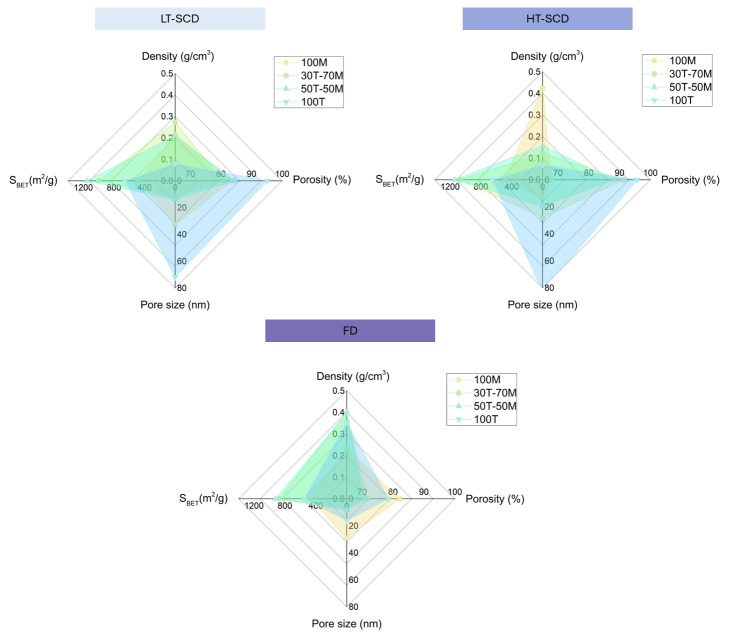
Summary of properties achievable by each drying method (low-temperature supercritical drying, high-temperature supercritical drying, and freeze-drying) depending on the surface’s hydrophilic/hydrophobic balance of silica gels.

When comparing the freeze-dried samples, aerogels produced with MTMS presented a more random and less linked network due to the presence of methyl groups not available for linking; thus, larger pores were expected [47,48]. However, it should be noted that, for having all samples with the same synthesis conditions, the procedure for obtaining MTMS gels was not optimal [35], which may have originated in higher-density gels. Furthermore, there were some synergistic effects related to the hydrophobicity of the MTMS structure. On the one hand, the reduced -OH groups present in 100MTMS decreased the interaction between the silica matrix and the ice crystals, therefore minimizing the subsequent shrinkage that occurred during sublimation. On the other hand, the inherent flexibility of the MTMS matrix allowed for better shrinkage withstanding. Both effects led to a minimized pore reduction, which is in agreement with the observed trend in freeze-drying shown in Figure 6. Moreover, when observing the 100M sample by SEM, it is obvious that it has a more fibrous-like structure than the colloidal-like gels of the other images. This effect may be due to some coarsening of the structural units caused by water aging and/or compression by the ice crystals.

Aerogels dried by LT-SCD and HT-SCD followed a similar trend between them, reaching the largest pores for the most hydrophilic sample (100T). This, even being contrary to what was expected for the chemical nature of the silica network with a high crosslinking through the -OH groups, also agreed with the density values, since a remarkably lower shrinkage was observed for this sample, leading to the presence of larger pores and overall porosity.

Considering the changes observed in the pore size as a consequence of the drying method and hydrophobicity of the silica matrix, the specific surface area of these materials was also expected to show significant modifications. As depicted in Figure 5b, the formulations containing both precursors (30T-70M and 50T-50M), which presented a lower hydrophobicity than 100M, showed higher surface areas with values above 1000 m^2^/g when dried by SCD methods. Nevertheless, these values were slightly reduced when applying freeze-drying.

Despite the conclusion that freeze-drying induced high densities in hydrophilic samples (100T), its effect was not remarkable on the specific surface area as a result of the balance between densification and the pore size reduction. The specific surface area reached for the 100MTMS sample was the same for the three different drying methods, providing evidence of the influence of the hydrophobic surface.

The obtained results demonstrated that, depending on the existence of polar (hydroxyl) or non-polar (methyl) groups on the gel surface, a different surface energy distribution would be achieved that depended on the drying method [41]. In the case of freeze-drying (see Figure 6, bottom), the interaction between water and the polar groups of the silica gel promoted structural densification, leading to densities ranging from 0.235 to 0.398 g/cm^3^. This contributed to reducing porosity with values between 70 and 82%, as well as inducing pore collapse, reaching the smallest pores (from 7.7 to 29.1 nm). Nevertheless, the effect on the specific surface area was not strongly remarkable, ranging from 479 to 922 m^2^/g.

However, for samples dried by SCD (LT and HT) (Figure 6 upper), it can be observed how the densities reached were lower (normally below 300 g/cm^3^), thus showing higher porosities (reaching a maximum of 95%), especially for samples presenting a more hydrophilic behavior. Furthermore, although pores were slightly larger than those obtained by freeze-drying, the specific surface areas were larger, achieving values as high as ca. 1150 m^2^/g.

It should be taken into account that the most hydrophobic sample (100MTMS) reached lower densities when dried by LT-SCD, with a value of 0.215 (g/cm^3^), than with HT-SCD (0.423 g/cm^3^), which could be explained by a possible further condensation and cluster formation happening at high temperatures.

## 3. Conclusions

The synthesis of silica gels with different hydrophobicity degrees was achieved by modifications in the precursor concentrations (TEOS and MTMS). Then, the effect of the drying method on the final textural properties and morphology (density, porosity, specific surface area, and pore size) was studied by following three procedures: freeze-drying, high-temperature supercritical drying with ethanol, and low-temperature supercritical drying with carbon dioxide.

The results reveal that hydrophobicity plays a key role in minimizing structural damage during drying, particularly under freeze-drying conditions, where more hydrophobic samples exhibited reduced shrinkage and better structural preservation. In contrast, HT-SCD led to increased densification (reaching densities of 0.423 g/cm^3^ for the 100M sample) in highly hydrophobic gels, likely due to further condensation reactions occurring at elevated temperatures. LT-SCD proved to be the most effective drying method for preserving pore structure in hydrophobic samples, leading to low-density (0.081–0.215 g/cm^3^), high-porosity (81–95%) aerogels.

Overall, this work provides critical insights into how the interplay between surface wettability and drying conditions can be leveraged to tailor the properties of silica aerogels, enabling optimized design for targeted applications in thermal insulation, adsorption, or catalysis. Most applications require thorough control of the aerogel structure to obtain optimum features such as smaller pores (below 100 nm to promote and effective Knudsen effect) for enhanced thermal insulation [49] and an increased specific surface area and porosity [50,51] and/or superhydrophobicity performance for oil/water separation [15]. Thus, these findings also emphasize the importance of considering both the chemical nature of the silica matrix and the thermal conditions of the drying process to achieve aerogels with desirable performance.

## 4. Materials and Methods

### 4.1. Materials

The silica precursors were TEOS (tetraethyl orthosilicate; Si(OC_2_H_5_)_4_; Acros Organics, Geel, Belgium), and MTMS (trimethoxymethylsilane; CH_3_Si(OCH_3_)_3_; Aldrich, Milwaukee, WI, USA). The acid catalyst for the sol–gel chemistry was oxalic acid (C_2_H_2_O_4_; 99%; Fluka Analytical, Fluka Chemie GmbH, Buchs, Switzerland) as a 0.01 M aqueous solution, and the basic catalyst was ammonium hydroxide (NH_4_OH, 25% NH_3_ in H_2_O, Fluka Analytical) as a 2.5 M aqueous solution. Ethanol (EtOH, absolute, C_2_H_5_OH), employed as a solvent, was supplied by Fluka Analytical. All reagents were used without further purification steps.

### 4.2. Synthesis of the Silica Aerogels

The corresponding amount of the silica precursor (TEOS and MTMS) was diluted in ethanol (10 mL of solution for each sample). Then, the two-step sol–gel process was initiated by adding the acidic catalyst (oxalic acid 0.01 M), and the mixture was stirred at 27 °C and 300 rpm for 30 min. Then, the sealed solution was placed in an oven for complete hydrolysis at 27 °C for 24 h, following the procedure described by Lamy-Mendes et al. [52]. After this step, the basic catalyst was quickly dropped into the mixture and stirred at 300 rpm for 15 s. Once a permanent gel was obtained, i.e., with no flowing when tilting the container, the gel time was taken, and the gels were placed in an oven at 27 °C for 5 days for ageing. Finally, gels were dried by the different methods explained in the next section.

**Figure 7:**
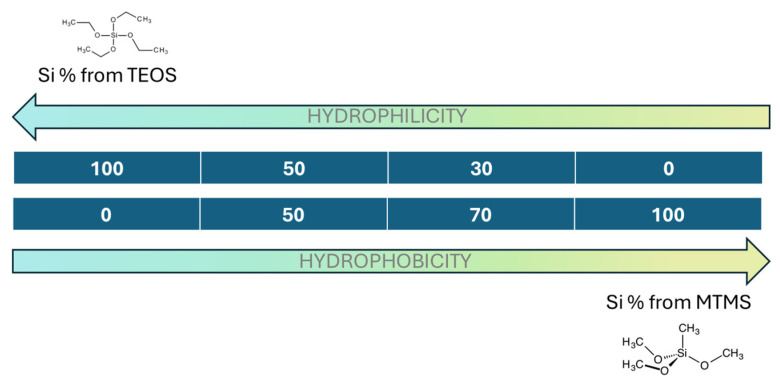
Different formulations developed in this work and their water affinity prediction.

Four different formulations were developed by modifying the amounts of both precursors (TEOS and MTMS). The molar ratio of Si in the network was kept as 100% TEOS (sample labeled as 100T), 50%TEOS-50%MTMS (labeled as 50T-50M), 30%TEOS-70%MTMS (labeled as 30T-70M), and 100% MTMS (labeled as 100M), as described in Figure 7. The catalyst (acidic and basic) amount was held constant for proper comparison, as well as the hydrolysis, condensation, and aging conditions. The volumetric ratio between ethanol, TEOS, MTMS, acidic and basic catalysts, and the gel volume was 0.62:0.23:0:0.08:0.08 for 100T, 0.64:0.12:0.08:0.08:0.09 for 50T-50M, 0.65:0.07:0.11:0.08:0.09 for 30T-70M, and 0.67:0:0.16:0.08:0.09 for 100M. Two replicates were produced for each formulation and drying method.

### 4.3. Drying Methods

The drying conditions for the low-temperature supercritical drying (LT-SCD) with carbon dioxide were 140 bar and 50 °C, with a previous washing of unreacted species by ethanol at RT. When gels were dried by high-temperature supercritical drying (HT-SCD), ethanol was employed as fluid, and the drying conditions were 80 bar and 260 °C. Freeze-drying was also employed as a drying technique at 19 Pa and −82 °C with a freeze-dryer model FDL-LON-80-TD-MM. Prior to this drying, samples were submitted to a solvent-exchange process by replacing the ethanol from pores with distilled water (4 × 12 h, RT).

### 4.4. Characterization Methods

#### 4.4.1. Bulk Density, Solid Density, and Porosity

Bulk density (*ρ*_B_) was obtained as the ratio between mass and geometrical volume, as described in ASTM D1622/D1622M-14 [53]. The solid density (*ρ*_s_) of the silica aerogels was determined by helium pycnometry with an AccuPyc II 1340, Micromeritics at 19.5 psi. Porosity was calculated by Equation (1):

(1) $$\Pi =\left(1-\rho_{r}\right)*100$$

where *ρ_r_* is the relative density obtained as the ratio between the bulk density and the solid density.

#### 4.4.2. Contact Angle

The sessile drop method was employed to determine the hydrophobicity degree by suspending a 15 µL droplet of ultra-pure water on the sample surface [54]. The contact angle measurements from the photographs taken were carried out with ImageJ software 1.50a.

#### 4.4.3. Specific Surface Area (*S_BET_*) and Pore Size

The specific surface area (*S_BET_*) was measured by nitrogen sorption with a Micromeritics ASAP 2020 instrument in the University of Málaga (Spain). Samples were first degassed under vacuum at 25 °C for 24 h, and then experiments were carried out at 77 K in the range *P*/*P*_0_ = 0.05–0.30. The specific surface area was obtained using the Brunauer–Emmett–Teller (BET) method [55].

From this value, the mean pore size was calculated using Equation (2):

(2) $$\Phi_{pore\;}=\frac{4V_{p}}{S_{BET}\;}$$

where *V_p_* is the total pore volume, which was determined as the gaseous volume per unit of mass by subtracting the skeleton volume (1/*ρ_s_*) from the total volume (1/*ρ_B_*) of the monolith, as described in Equation (3):

(3) $$V_{p}=\frac{1}{\rho_{B}}-\frac{1}{\rho_{s}}$$

#### 4.4.4. Morphology Observation by Scanning Electron Microscopy (SEM)

The nanoporous structure of silica aerogels was assessed using a field-emission scanning electron microscope, FESEM (Compact/VP Compact FESEM, from Zeiss Merlin, Zeiss, Oberkochen, Germany). Before the visualization, samples were gold-sputtered for suitable electrical conductivity.

## Figures and Tables

**Table 1:** The main structural properties of the obtained aerogels, classified by formulation and the drying method.

Sample	Bulk Density (g/cm^3^)	Porosity (%)	*S*_BET_ (m^2^/g)	Mean Pore Size (nm)
	LT-SCD			
100T	0.081	95.1	655.1 ± 1.2	71.82
50T-50M	0.211	84.4	1153.4 ± 3.4	13.9
30T-70M	0.272	80.7	998.7 ± 2.0	11.9
100M	0.215	83.7	477.1 ± 3.1	32.6
	HT-SCD			
100T	0.071	95.7	652.4 ± 1.7	82.6
50T-50M	0.159	88.3	1141.9 ± 3.7	19.5
30T-70M	0.123	91.3	1064.6 ± 2.4	27.9
100M	0.423	67.8	478.8 ± 2.3	13.4
	Freeze-drying			
100T	0.353	78.5	551.5 ± 1.1	16.1
50T-50M	0.397	70.6	922.6 ± 3.5	7.7
30T-70M	0.398	71.7	872.4 ± 2.6	8.3
100M	0.235	82.1	479.8 ± 2.2	29.1

## Data Availability

Data will be available on request.
